# Rapid Near-Patient Impedimetric Sensing Platform for Prostate Cancer Diagnosis

**DOI:** 10.1021/acsomega.4c00843

**Published:** 2024-03-15

**Authors:** Parisa Dehghani, Vaithinathan Karthikeyan, Ataollah Tajabadi, Dani S. Assi, Anthony Catchpole, John Wadsworth, Hing Y. Leung, Vellaisamy A. L. Roy

**Affiliations:** † James Watt School of Engineering, University of Glasgow, Glasgow G12 8QQ, U.K.; ‡ Scottish Trace Element and Micronutrient Diagnostic and Research Laboratory, Department of Biochemistry, Royal Infirmary, Glasgow G31 2ER, U.K.; § Cancer Research UK Scotland Institute, Glasgow G61 1BD, U.K.; ∥ School of Cancer Sciences, MVLS, University of Glasgow, Glasgow G61 1BD, U.K.; ⊥ School of Science and Technology, Hong Kong Metropolitan University, Ho Man Tin, Hong Kong

## Abstract

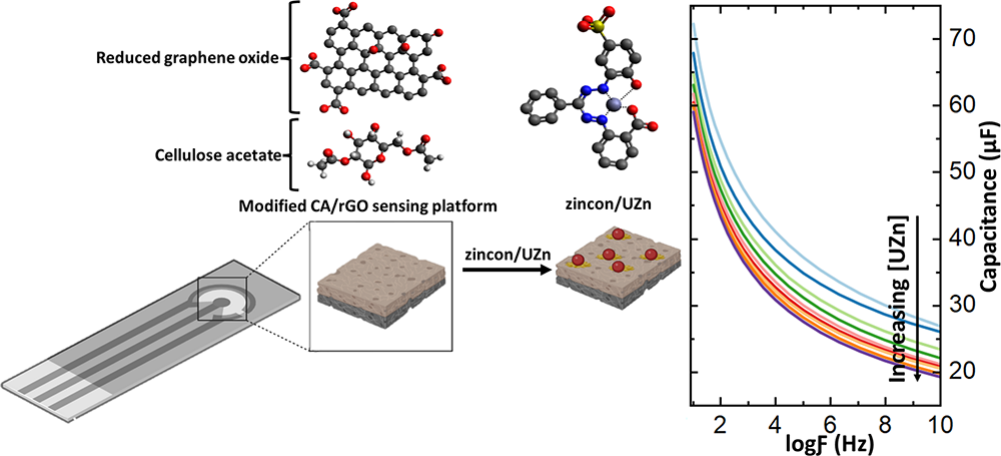

With the global escalation of concerns surrounding prostate cancer (PCa) diagnosis, reliance on the serologic prostate-specific antigen (PSA) test remains the primary approach. However, the imperative for early PCa diagnosis necessitates more effective, accurate, and rapid diagnostic point-of-care (POC) devices to enhance the result reliability and minimize disease-related complications. Among POC approaches, electrochemical biosensors, known for their amenability and miniaturization capabilities, have emerged as promising candidates. In this study, we developed an impedimetric sensing platform to detect urinary zinc (UZn) in both artificial and clinical urine samples. Our approach lies in integrating label-free impedimetric sensing and the introduction of porosity through surface modification techniques. Leveraging a cellulose acetate/reduced graphene oxide composite, our sensor’s recognition layer is engineered to exhibit enhanced porosity, critical for improving the sensitivity, capture, and interaction with UZn. The sensitivity is further amplified by incorporating zincon as an external dopant, establishing highly effective recognition sites. Our sensor demonstrates a limit of detection of 7.33 ng/mL in the 0.1–1000 ng/mL dynamic range, which aligns with the reference benchmark samples from clinical biochemistry. Our sensor results are comparable with the results of inductively coupled plasma mass spectrometry (ICP-MS) where a notable correlation of 0.991 is achieved. To validate our sensor in a real-life scenario, tests were performed on human urine samples from patients being investigated for prostate cancer. Testing clinical urine samples using our sensing platform and ICP-MS produced highly comparable results. A linear correlation with *R*^2^ = 0.964 with no significant difference between two groups (*p*-value = 0.936) was found, thus confirming the reliability of our sensing platform.

## 1 Introduction

Prostate cancer (PCa) ranks as the second most common cancer diagnosed in men worldwide.^1^ Accurate identification of invasive PCa continues to be a critical clinical need due to the danger of premature death from PCa and the lack of specific symptoms in the early stage of the disease. The increasing and aging global population is driving the demand for cost-effective prostate cancer diagnostic tools that can be readily accessed by various healthcare institutions. There is increasing interest in applying novel biophysical methods to support early detection of PCa. The current clinical gold standard methods for diagnosing PCa include digital rectal examination (DRE) and the measurement of serum prostate-specific antigen (PSA). The definitive confirmation of tumor presence is achieved through the conduction of transrectal ultrasound-guided prostate biopsy (PB).^2,3^ However, the serum PSA level, typically set at >4 ng/mL, is not a definitive indicator of PCa, as it can be influenced by irritations, prostatitis, benign prostatic hypertrophy (BPH), or dietary factors.^4−6^ Consequently, PB is crucial for a conclusive diagnosis. Nevertheless, PB is an invasive procedure and exhibits low accuracy, especially during the initial biopsy, particularly in the early stages of the disease.^7^ Consequently, the need for repeated biopsies arises, causing discomfort to the patient and contributing to overtreatment, along with imposing additional costs on national health systems.^6,8^ Therefore, there is a need for a wider array of diagnostic biomarkers. Among the many biomarkers, urinary zinc (UZn) is considered to be an excellent candidate because even at early (organ-confined) stages, malignant tissue cells exhibit discernible alterations in UZn content when compared to both healthy prostate tissue and benign prostatic hyperplasia (BPH).^9^ Normal prostate tissue reveals a large accumulation of zinc in the anatomical peripheral zone, which leads to a 10-fold higher concentration compared with other soft tissues. As a result, the concentration of zinc in urine reflects the levels of zinc in the prostatic tissue.^10,11^ The modulation of UZn levels appears to be the most significant in prostate cancer, allowing its differentiation from other common prostatic pathologies such as BPH and prostatitis.^12,13^ In a healthy context, UZn levels typically range between 200 and 400 ng/mL. Deviations from this range, as indicated by conflicting findings in the literature review, should be considered as potential indicators of a cancerous state.^9,14−22^ In-depth investigations and studies are being carried out to establish a clearer understanding of the underlying mechanisms and implications of zinc.^23−25^

In general, specialized equipment and associated support from skilled technicians are necessary for the detection of UZn by atomic absorption spectroscopy, inductively coupled plasma mass spectrometry (ICP-MS), and surface-enhanced Raman spectroscopy (SERS).^11,26−29^ Colorimetric analysis using reagents such as o-2-(2-hydroxy-5-sulfophenylazo) benzylidenehydrazinobenzoic acid was assessed as a convenient alternative means to detect UZn in patient samples.^30^ However, such convenient alternative methodologies tend to suffer from low specificity because of their inability to differentiate metal ions like Zn^2+^ and Cu^2+^ present in urine.^31^ In this context, electrochemical methodologies emerge as a promising avenue for UZn detection, presenting notable advantages such as precision, user-friendliness, heightened specificity, noninvasive protocols, and cost-effectiveness. Within electrochemical systems, bismuth and mercury electrodes have been widely employed for zinc detection.^32−36^ Nevertheless, the limited applicability of mercury because of its toxicity and bismuth due to the lack of biocompatibility and complexity of fabrication has resulted in reduced focus on their usage.^33−35^ In recent times, carbon-based materials, including carbon nanotubes, graphene, and carbon nanoparticles, have demonstrated exceptional sensitivity, achieving a low limit of detection (LOD) in the nanomolar range.^32,33,37^ However, despite their heightened sensitivity, these carbon-based materials exhibit low specificity when used individually. Consequently, their application for zinc detection in body fluids has infrequently been explored. To address this limitation, a promising strategy is modifying carbon-based materials using chemical molecules such as amino acids, EDTA, and zincon.^38,39^ These molecules have the potential to form complexes with zinc, enhancing the specificity of detection.^30,38−40^ At present, electrochemical techniques are employed for the purpose of carbon-based material functionalization with zincon,^41−44^ facilitating the electrochemical quantification of heavy metals, specifically Pb and Zn.^38,40^ This modification process requires to be done accurately to maintain the integrity of active sites and prevent them from blockages.^45,46^ Efforts have been made to detect zinc in body fluids, such as prostate fluid and urine, utilizing exfoliated graphite^38^ and carbon nanotubes (CNT)^39^ modified with zincon. However, the poor stability was coupled with a high LOD.^38,39^ The mentioned drawbacks emphasize the need for the development of a new alternative strategy. The objective of this strategy should be to ensure the sensitivity, stability, and reliability of the device for medical applications and potential commercialization.

This study introduces a groundbreaking approach by integrating label-free impedimetric sensing and strategically introducing porosity through surface modification techniques of screen-printed carbon electrodes (SPCE). Utilizing a cellulose acetate and reduced graphene oxide (CA/r-GO) composite, along with zincon as a receptor, the recognition layer of our capacitive sensing platform is engineered to exhibit enhanced porosity and selectivity, critical for capturing and interacting with UZn with high sensitivity. To the author’s knowledge, this work marks the first instance of an electrode being modified with this unique combination of materials. Here, the immobilization of zincon further amplifies sensitivity, establishing highly effective recognition sites and ensuring unparalleled detection accuracy. Furthermore, the label-free capacitive sensing platform employed in our study utilizes nonfaradaic impedimetric spectroscopy. This approach offers real-time monitoring of alterations in electrical double-layer capacitance resulting from the UZn–zincon interaction at the electrode/analyte interface. This combination of label-free impedimetric sensing, enhanced porosity, and external doping distinguishes our capacitive sensing platform from existing impedimetric biosensors for UZn detection, positioning it as a promising advancement in the field of PCa diagnostics. Also, the regeneration ability of the sensing platform extends the capabilities of the device beyond its immediate advantages, which opens up many avenues for future applications.

In the context of electrochemical double-layer (EDL) capacitors, electrode materials with specific attributes are highly sought after. These attributes include a high degree of porosity, pores accessible to the electrolyte, electrical conductivity, and cost-effectiveness.^15,19^ Furthermore, it is particularly advantageous for nonfaradaic capacitor electrodes to exhibit electrochemical inertness, a quality that positions carbon-based materials as exceptionally well-suited candidates for this purpose.^47,48^ Here, the CA/r-GO-modified layer exhibits superior characteristics such as higher surface area, conductivity, pores, and vacancies.^49−51^ These features provide ample opportunities for chemical interactions between the electrode and analytes, offering significant advantages for surface reactions and adsorption processes in sensing approaches. Furthermore, the modified layer demonstrates the potential to enhance the deposition tendency of zinc along the CA/r-GO crystal plane.^52^ We benchmark the performance of our sensor platform with established clinical standards using clinical biochemistry reference materials (CBR), followed by the analysis of clinical urine samples.

## 2 Theoretical Section

In electrochemical engineering and electrochemistry, faradaic and nonfaradaic processes that happen on the electrode’s surface are clearly distinct.^53,54^ In the faradaic process, charge transfer is the most predominant phenomenon that occurs in the conductive electrode/electrolyte interface. Here, charges should transfer and are not stored in the electrode. In a nonfaradaic (capacitive) process, electric charge gradually accumulates on the surface of the electrode. Moreover, in a nonfaradaic process, a binding interaction between ions and receptors occurs within the electrode structure.^55,56^ As a result, the nonfaradaic process effectively facilitates the storage of both ions and charges. Here, even after an oxidation/reduction reaction, the charged species do not leave the electrodes,^57,58^ for example, Li^+^ ions interacting with the surface are reduced after reaching the electrode and get stored as Li atoms. The charged species’ absorption within the electrode can alter the double-layer capacitance (CEDL).

The Helmholtz model can describe *C*_EDL_. Based on this model, the first layer at the electrolyte interface, where most of the counterions are held and immobilized, is the Helmholtz layer.^30^ Its thickness is supposed to be equivalent to the single-ion radius solvated in solution, typically restricted to nanometer scales. The subsequent topmost layer called the diffuse layer is characterized by considering the Poisson–Boltzmann relationship between the electrostatic interactions and diffusion effects of counterions.^59,60^ Diffuse layer electrical behavior can be likened to a separate capacitive element, determined by the electrolyte’s Debye length.^61^ Thus, both the diffuse layer and Helmholtz interfaces can be effectively described as discrete capacitive elements such as *C*_diffuse_ and *C*_Helmholtz_, respectively. Accordingly, *C*_EDL_ can be described as follows:
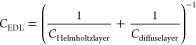
1

The behavior of carbon-based porous materials in *C*_EDL_ depends significantly on structural characteristics such as porosity and surface modification.^15,18^ Generally, by increasing the surface area in the porous structure, the capacitance is improved. To achieve this, chemical activation by NaOH or KOH is mostly used to develop the porosity and distribution of micropore sizes.^62^

## 3 Experimental Section

### 3.1 Material and Instruments

Clinical samples studied in this study were obtained from the NHS Research Scotland Greater Glasgow and Clyde Biorepository with consent and ethical approval under tissue bank no. REC 22/WS/0020. The SPCEs were purchased from Wellsun Limited (Kushan Development zone, China). Cellulose acetate (CA) (*M*_n_ ∼ 30,000), reduced graphene oxide (r-GO), dimethyl fumarate (DMF), zincon, methyltrioctylammonium chloride, ethylenediamine, zinc chloride, sodium hydroxide, and copper(II) chloride were purchased from Sigma-Aldrich. The purest grade of reagents is used for all commercially ordered reagents, and predistillation was not required before their use. The creatinine (enzymatic) reagent kit was purchased from Alinity. All of the glassware were purchased from Thermoscience. Allegra X-30 Centrifuge is used for preparing the clinical samples. A Phenom XL Benchtop SEM system (Thermo Fisher Scientific, USA) is used for SEM and energy-dispersive X-ray (EDX) imaging to characterize the structure and dimensions of electrode surfaces, as well as their chemical properties. For sputtering gold on the SPCE, after each step of modification with CA/r-GO and zincon before SEM and EDX analysis, an Emitech K550 gold sputter coater (PerkinElmer, Australia) is used. A Nicolet iS5 Fourier transform infrared (FTIR) spectrophotometer with attenuated total reflectance (ATR) accessory is used for FTIR analysis of the modified electrode. A Heidolph Reax top shaker vortex mixer (Heidolph, Germany) is utilized to uniformly mix samples. A Raman spectrometer is used to characterize the chemical structure on the surface of the electrode. Vortex and a hot plate are used for mixing the solutions and baking step, respectively.

### 3.2 Synthesis of the Cellulose Acetate/r-GO Composite

The CA solution was prepared by the dissolution of 12 wt % CA in DMF under continuous stirring. 0.5 g of NaOH/100 mL solution was added to the CA solution to enhance the number of hydroxyl groups on the surface of the polymer (treated CA). Through this treatment, acetyl groups in the polymer were hydrolyzed with NaOH, which increased the number of hydroxyl groups and facilitated the reaction between the OH groups of CA and OH or COOH groups of r-GO.^63,64^ r-GO at 1 mg/mL concentration in DMF was prepared and added to the CA solution in a 1:1 ratio. The concentrations of CA and r-GO were selected based on scientific literature reporting values high enough to prepare the polymeric matrix and improve/modify its features.

### 3.3 Electrode Modification with CA/r-GO Composite

**Figure 1:**
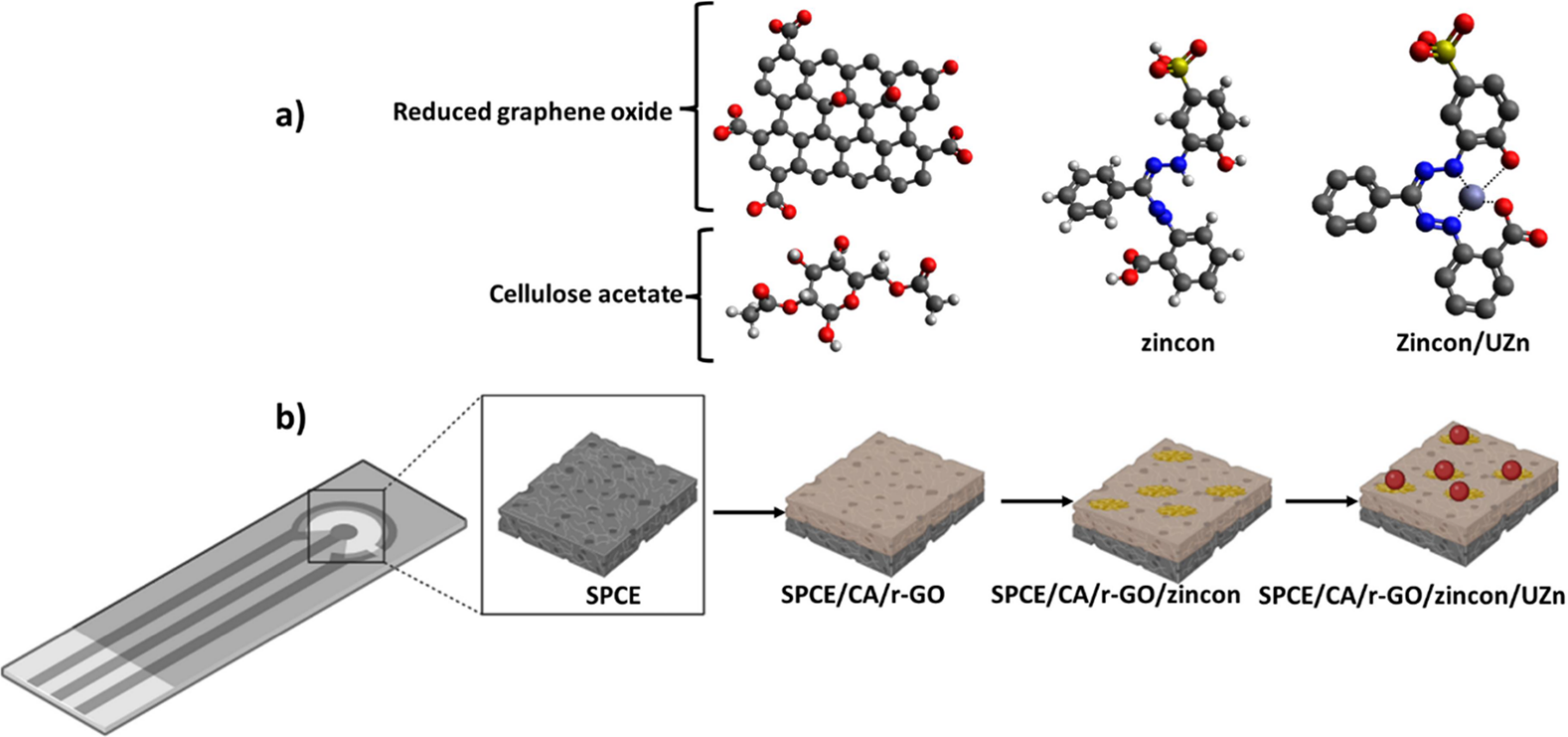
Schematic images of the (a) chemical structure and interaction between CA/r-GO and zincon/UZn and (b) surface modification process of modified sensing platform with the CA/r-GO composite, zincon, and introducing UZn solution.

The SPCE was cleaned in 0.1 M H_2_SO_4_, using CV (−1.5 V to 0 for 10 cycles), washed with DI water, and dried under N_2_ gas. Afterward, the CPE was preanodized in 0.05 M PBS (pH 7.4), by applying 1.7 V for 3 min, before being washed and dried under N_2_ gas. After preanodizing, the impedance value decreased significantly, which is a sign of minimum contamination of the surface of the electrode, as shown in Figure S4a. Finally, 10 μL of the CA/r-GO suspension was drop-casted on the surface of the SPCE working electrode and baked overnight at 30 °C to evaporate DMF. The schematic diagram of electrode modification is shown in Figure 1b.

### 3.4 Urinary Zinc Detection

By mixing 1 g of zinc chloride powder with 1,000 mL of artificial urine (EN 1616:1999, explained in Supporting Information), a concentrated stock solution containing 1000 μg/mL UZn is prepared and refrigerated for future use. To prepare the samples, serial dilution is used. Various samples with varying concentrations of UZn are tested with pure artificial urine with a zero level of UZn. The pH of various concentrations of Zn salt solutions was adjusted to pH 9.8 by adding an ammonia/ammonium chloride buffer, considering the highest molar absorption coefficient of Zn to zincon.^65−67^ For each concentration of UZn, 10 μL of the solution was introduced on the surface of the modified sensing platform and left for 10 min at 25 °C to react. Following that, the modified sensing platform was washed with DI water to minimize some nonspecific binding and eliminate extra UZn, which did not adsorb to the zincon. Similarly, 437.39, 625.03, and 851.90 ng/mL of CBR samples from the Scottish Trace Element and Micronutrient Diagnostic and Research Laboratory are prepared to validate the data by ICP-MS. The pure blank sample with a zero level of UZn is used as a control solution.

### 3.5 Electrochemical Impedance Spectroscopy Measurement

Electrochemical impedance spectroscopy (EIS) was used with an Autolab instrument controlled by NOVA for UZn detection. The measurement was done at 25 °C to enervate the effect of temperature on the performance of the modified sensing platform. The SPCE consists of three electrodes: a working electrode on which all immobilization and modification were applied (3.2 mm^2^), a reference electrode, and a counter electrode. During the measurement, stirring was not performed. The range of frequency for EIS was adjusted between 10 mHz to 10 kHz with an amplitude of 0.1 V in a 0.05 mol/L K_3_[Fe (CN)_6_]/K_4_[Fe (CN)_6_] buffer solution to improve the capacitance.^68−70^ The experiment is repeated three times.

### 3.6 Regeneration Process

Electrochemical chronoamperometry was used for the one-step regeneration process. Here, −1.2 DC voltage was applied for 5 min to the surface of the modified sensing platform, which was occupied with UZn, in PBS 0.05 M (pH = 7.4). The surface of the modified sensing platform was washed with DI water and kept at room temperature to dry.

## 4 Results and Discussion

### 4.1 Development of the Binding Surface

The state-of-the-art electrochemical differential pulse voltammetry method suffers from the overlapping reduction potentials of Zn^2+^ and H^+^ ions (around −1.2 V) to detect UZn, thus masking the authentic signals from Zn^2+^ ions.^38^ Furthermore, the same valence ions such as Cu^2+^ compete over the binding sites, and as a result, the electrochemical detection peak for UZn is suppressed. To overcome these obstacles, surface-engineered electrodes with exclusive ion selectivity are essential for electrochemical detection. To minimize off-target (nonspecific) signals from other metal ions, we modified the electrodes for ion detection by immobilizing zincon using CA and a r-GO thin film coated with methyltrioctylammonium ion and conducted the experiment at pH 9.8. The CA/r-GO interface layer provides a binding surface for π–π interactions^38,71^ and covalent binding^39^ between the CA/r-GO layer and zincon with good conductivity for readout as electrochemical signals. Besides, the porous structure maximizes the mass transport of analytes and reagents. The chemical formula and electrode deposition process are illustrated in Figure 1a,b, respectively.

### 4.2 Morphology Characterization

**Figure 2:**
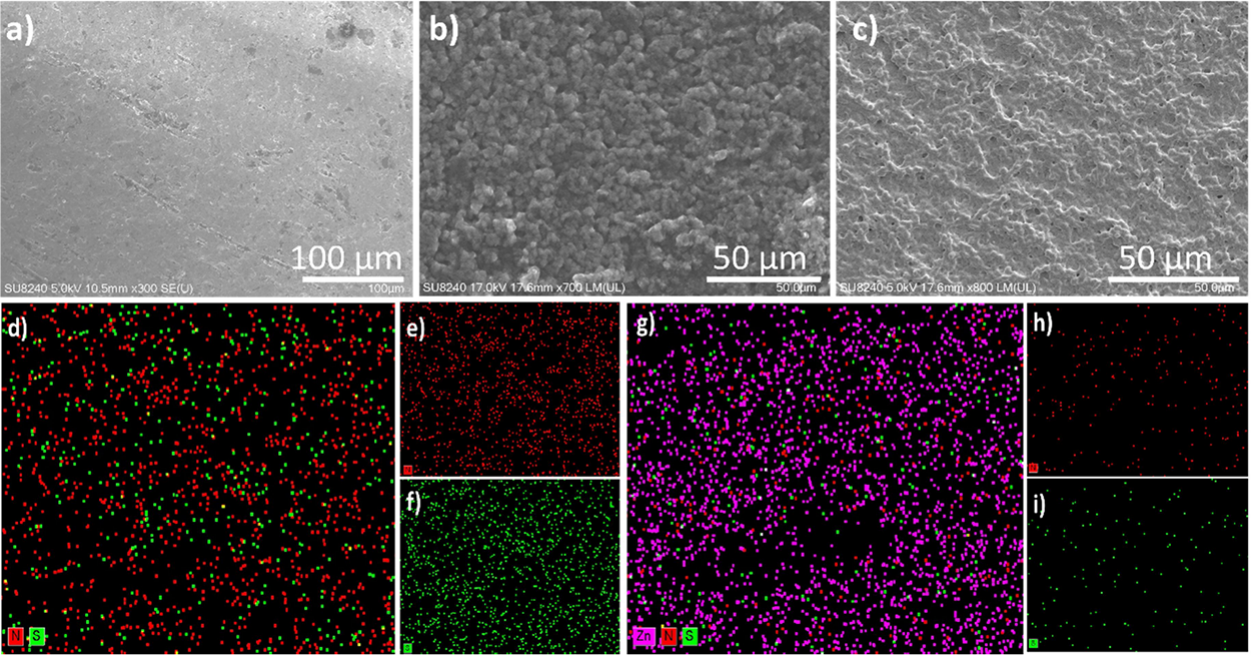
SEM image of the modified sensing platform without (a), with CA/r-GO modification (b), and after introducing zincon functionalization on the surface of the modified sensing platform (c). EDX analysis of the porous modified sensing platform with zincon (d), N (e) and S (f) elements individually and following the addition of UZn to the surface of the modified sensing platform (g). Also, the reduction in density of N (h) and S (i) elements is observed after the introduction of UZn.

Figure 2 shows the SEM images of surface morphology and the development of structural features after each step of modification, while introducing UZn on the modified sensing platform. The SEM pictures clearly demonstrate the development of porous surface morphologies following the CA/r-GO composite coating, which maximizes the mass transfer of analytes and UZn to the surface of SPCE. It effectively communicates that the modification of the CA/r-GO electrode introduces a porous structure, leading to a significant difference when compared to the bare counterpart. The cross section of the modified sensing platform after surface functionalization with zincon is presented in Supporting Information in Figure S1. It highlights the distinct 3D porous structure with interconnected pores observed in the cross-sectional image. The evidence of the deposited thin film material is seen in the EDX spectrum showing the elemental mapping of N and S from zincon and UZn over the modified sensing platform. The atomic presence of N and S after adding zincon to the surface of the modified sensing platform is shown in Figure 2c. According to EDX spectra, the modified sensing platform with zincon is confirmed by the presence of nitrogen and sulfur elements in the zincon chemical structure (Figure 2d–f). The EDX graphic spectra of the modified SPCE with zincon confirm the presence of two nitrogen and sulfur elements which are the two strategic atoms in the zincon chemical structure. As shown in Figure 2g, after introducing the UZn solution to the surface of the modified sensing platform, the fraction of N and S groups is dramatically reduced, indicating that the metal receptors (zincon) on the surface of the modified sensing platform interacted with the target (UZn) (Figure 2h,i).^39,72,73^ Collectively, data from SEM and EDX successfully validate the porous structure of the modified CA/r-GO layer and the interaction between zinc oxide and UZn.

Atomic force microscopy (AFM) is employed to scrutinize the surface morphology postzincon modification and subsequent exposure to UZn (Figure S3). In this analysis, the root-mean-square (RMS) roughness increases to 9.9 nm due to external doping induced by zincon. However, following the introduction of UZn, the RMS decreased from 9.9 to 1.7 nm. This reduction is attributed to the interaction between zincon and UZn, as well as ion accumulation on the surface of the modified SPCE, leading to a reduction in porosity.^74,75^

### 4.3 Surface Modification Process of the Modified Sensing Platform

To further investigate the surface modification and interaction between the layers of CA/r-GO functionalized with zincon and UZn sites, we performed FTIR spectroscopy (Figure S2a). The presence of broadband peaks observed at 678 and 1523 cm^–1^ is associated with ≡C–H bonding and C–N deformation, indicating the bonds of zincon-functionalized CA/r-GO/SPCE, respectively. The peak at 1619 cm^–1^ corresponds to C=C bonds and confirms the π–π interaction between zincon and CA/r-GO/SPCE, while peaks between 1200 and 1250 cm^–1^ are attributed to C–O. FTIR peaks at 2844 and 3275 cm^–1^ correspond to zincon and are associated with C=CH and OH in the carboxylic acid group; however, these characteristic peaks become imperceptible within the SPCE-CA/r-GO-modified layer after the introduction of zincon.^39^ X-ray diffraction (XRD) analysis was carried out to identify and demonstrate the structural interaction of the CA/r-GO composite with zincon and, subsequently, the modified CA/r-GO/zincon with UZn. The observed peak at 26.6° represents a highly ordered crystal structure (002) of graphene with a basal spacing of 3.4 *A*°. The XRD peak at 9.07° represents the basal spacing resulting from the oxidation groups of r-GO.^76^ It can also be observed that the intensity of these peaks at 38.3° decreases because of the zincon functionalization of CA/r-GO. The effect of UZn introduced on the porous surface can be clearly seen by the fading of the 18.2° peak, which corresponds to the (002) crystal surface of carbon (Figure S2b).

### 4.4 Urinary Zinc Detection via a Nonfaradaic Approach and EIS Technique

EIS signals can be investigated as two different types: faradaic and nonfaradaic, as a result of selective interactions at the surface of the electrode within the recognition layer. In the faradaic process, detection is based on the charge-transfer signal resulting from the oxidation/reduction reaction of redox molecules occurring at the electrode/analyte interface of the biosensor.^76−78^ On the other hand, capacitive and resistive alterations at the interface layer, due to selective interaction between the target and receptor and ion accumulation, are predominant phenomena in nonfaradaic sensing platforms. Therefore, nonfaradaic, as a label-free process, does not require low voltage for perturbation, which is a valuable feature for point-of-care devices.

Based on eq 1, *C*_EDL_ is the sum of *C*_Helmholtz_ and *C*_Diffusion_ which are placed in the series. At the electrode–analyte interface, *C*_EDL_ occurs due to the accumulation of electrostatic charges. In general, the faradaic performance is more sensitive than the capacitive process. An innovative approach to enhance the sensitivity of the capacitance process involves increasing the surface area by introducing a porous structure and creating electrochemical supercapacitor electrodes. Here, activating the platform with large micropores would enhance *C*_EDL_ by improving the analyte’s accessibility to the pores (eq 2).^59,62^
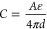
2where *A* is the active porous surface area; the medium dielectric constant is presented by ε, which is related to the analyte; and the electrochemical double layer thickness is shown by *d*.

Therefore, our sensing platform demonstrates remarkable success by exhibiting an ultrahigh unit area capacitance (UAC), reaching magnitudes of microfarads per square centimeter (μF cm^–2^).^19^ This is attributed to the surface modification with the CA/r-GO composite, which creates a porous structure and increases the surface area, leading to the enhancement of *C*_EDL_ as shown in Figure S4. The modified porous SPCEs such as our CA/r-GO/SPCE typically exhibit limited utilization of their surface area due to the presence of inaccessible deep and branched pores.^20^ Additionally, they demonstrate a notably low intrinsic contribution to capacitance per unit surface area, known as the specific areal capacitance. As a result, merely increasing the surface area yields only modest improvements in capacitance, while the low packing density of the CA/r-GO/SPCE porous sensing platform further hinders achieving the desired volumetric capacitance necessary for device miniaturization. To address this limitation, the surface of the modified porous SPCE was functionalized by zincon, which acts as an external doping. The formation of a recognition layer by the immobilization of zincon significantly enhanced the capacitance of CA/r-GO/SPCE, thereby overcoming the aforementioned challenges and offering the potential for satisfying the demands of miniaturized devices (Figure S4b).

Quantitatively, *C*_EDL_ can be considered as a combination of 1/*C*_Helmholtz_ and 1/*C*_Diffusion_. Here, the thickness of the diffuse layer, called the Debye length, represents the distance where the electric field is screened. Therefore, to improve sensitivity, the interaction between the target and the recognition layer should occur within this region, which is approximately 1 nm.^79−81^ However, based on the literature review, the estimated length of zincon is small enough (less than 500 Å) to enable the interaction between UZn and zincon to occur inside the Debye length.^82^ According to the results, *C*_EDL_ is substantially enhanced in the modified porous SPCE functionalized with zincon. This improvement directly enhances the sensitivity of the sensing platform, aligning well with theoretical predictions.

**Figure 3:**
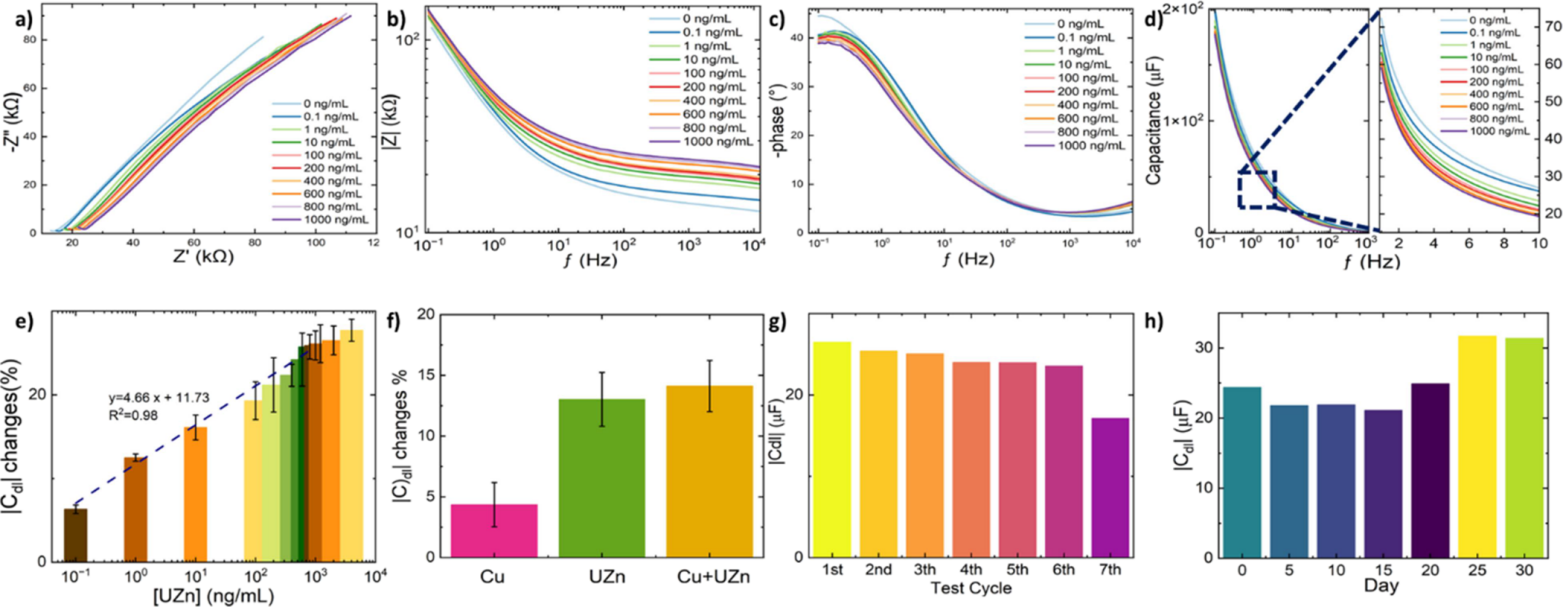
EIS measurement of different concentrations of UZn from 0.1 to 1000 ng/mL for 10 mHz to 10 kHz range of frequency, when applying 10 mV input voltage. (a) Nonfaradaic Nyquist plot, Bode plot (b), phase plot angle indication of the alteration in both resistive and capacitive domains, at high frequencies toward low frequencies (c) and capacitance vs frequency (d). The nonlinear calibration curve (e) is illustrated; also, the inset image presents the calibration curve of the normalized value of different UZn levels in lab-based samples in its linear range. Selectivity of the sensing platform during the differentiation between Cu^2+^, Zn^2+^, and the mixture solution of Cu^2+^/Zn^2+^ (f) and the repeatability of the modified sensing platform after running EIS measurement seven times (f) are demonstrated. All measurements are rigorously executed at a pH of 9.8. This specific pH setting is meticulously selected due to its alignment with the optimal pH range for the UZn/zincon complex, spanning from 9.5 to 9.8. Within this range, the complex exhibits its peak performance in terms of effective molar absorption coefficients, rendering it ideal for our analytical purposes. Repeatability (g) and stability (h) of the modified porous electrode are presented.

To determine the ratio and concentration level of UZn in the urine samples, interacted UZn on the modified sensing platform was analyzed using the EIS approach. Figure 3a illustrates the impedance Nyquist plot for different target concentrations ranging from 0.1 to 1000 ng/mL for the 10 mHz–10 kHz range. Due to the nonfaradaic nature of the EIS pots, and detection procedure, the binding between zincon and UZn can be investigated by studying the *C*_EDL_ modulation. The interaction and capture of UZn can be elucidated by studying the alterations in *Z* imaginary (−*Z*”) (Figure 3a–d).^83^ Here, electrical charges are stored at the surface and/or inside pores, relying on double-layer charging. Therefore, the charging process in a porous SPCE is due to charge adsorption and accumulation at the interface between the electrode and the electrolyte, giving rise to *C*_EDL_. This process is largely electrostatic and nonfaradaic in nature. The presence of a negligible resistive charge-transfer component is indicated by the absence of a semicircle and small alterations in the Nyquist curves observed at high frequencies along the *Z*_real_ axis. It means that ideally no electron transfer takes place across the electrode/electrolyte interface.^84,85^ In addition, the absence of a redox label in a Nyquist plot represents that the reduction process did not take place; hence, charge transfer did not occur.^54^ Also, typical diffusion did not happen due to the slow transfer of electrons, which resulted in an incomplete semicircle Nyquist graph.^17,79,86,87^ In the low-frequency regime, the imaginary component of impedance is observed to be predominant. Therefore, the primary contributor to the imaginary part of impedance in this context is *C*_EDL_. The transient accumulation of charges resulting from UZn binding at the electrical double layer brings about a modification in the relative dielectric permittivity, contributing to the alteration in *C*_EDL_. *C*_EDL_ exhibited a decreasing trend with an increase in UZn concentration, which validates the hypothesis that the predominant factor contributing to the change in impedance across multiple UZn concentrations is the double-layer capacitance.^88^

EIS can provide the relationship between |*Z*| and frequency (*f*) which is called the Bode plot (Figure 3b). Therefore, eq 3 can be used to calculate the capacitance:
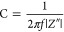
3

The Bode plot illustrates the reduction in capacitance as the frequency increases (Figure 3b). At high frequencies, the capacitors can behave like short circuits due to the limitations of ions entering the pores in the electrolyte and the formation of dielectric capacitance.^59,89^ Obviously, the magnitude of |*Z*| is increased by elevating the levels of UZn, which is particularly noticeable in the low-frequency region of the spectrum. This performance is anticipated for a capacitive sensing platform functioning on the basis of charge accumulation.

As depicted in the phase plot (Figure 3c), at low frequencies, the maximum values of the phase angle ranging from 30 to 40° correspond to the capacitive behavior of the modified porous SPCE associated with *C*_EDL_. However, as the phase angle approaches zero, the behavior of the sensing platform exhibits a more resistive nature. Additionally, it is observed that with an increase in the concentration of UZn, the phase angle decreases. This observation suggests that an increase in concentration leads to an enhancement in impedance at the electrode/electrolyte interface, attributed to the binding interaction between UZn and zincon. The negative phase angle indicates *C*_EDL_ change due to UZn and zincon interaction within the modified porous carbon electrode.^85,90−94^ Therefore, our nonfaradaic impedimetric modified porous SPCE functionalized with zincon demonstrates the capability to detect UZn quantitatively.

The alteration in electrical charge due to the binding between the receptor and target, the structure of the electrical double-layer, and the EIS-related parameters can be modeled and visualized by a customized version of the modified equivalent Randle’s circuit.^92^ In addition, a solution resistance (*R*_s_) is represented by the intercept on the *Z*′ (real) axis including the resistance of the sensing platform, wires, the ionic resistance of the target solution, and the inherent resistivity of the electrodes. As the modified porous SPCE operates on a label-free, nonfaradaic principle, the reduction process did not take place. Consequently, charge transfer did not occur dominantly, as depicted in Figure 3a.^54^ Hence, the parameters correlated to electron transfer and corrosion in our model such as charge-transfer resistance (*R*_ct_) and Warburg resistance (*W*) were eliminated.^17,79,86,87^

From EIS plots, the conductivity of the porous SPCE and electrolyte is indicated at low frequencies (<100 Hz). Therefore, at frequencies between 10 and 100 Hz, the electrode porousness and the capacitive behavior of porous SPCE can be studied.^59^ Consequently, charge perturbation is created at the surface of the electrode when UZn ions are captured by zincon. This can be a result of the UZn/zincon complex within an electrical double-layer, which leads to changes in *C*_EDL_. These changes alter the dielectric permittivity; therefore, a variation in *C*_EDL_ can be directly attributed to the UZn concentration change.^95,96^ This alteration is used to investigate the calibration curve (Figure 3e). By increasing the concentration of UZn, *C*_EDL_ is decreased because of ion adsorption on the surface of the sensing platform, which leads to a decline in the interfacial dielectric constant of EDL (Figure 3d).^97,98^

According to the AFM results, adding UZn reduces RMS roughness (Figure S3) and decreases the porosity, which lowers the penetration effectiveness of the electrolyte solution into the modified layer and increases *C*_EDL_, attributed to the interaction between zincon and UZn.^96^Figure 3e illustrates the calibration curve of UZn measured with the modified sensing platform for lab-based samples. The impedance responses of our device to different concentrations of UZn are normalized using the baseline (100 × Δ*C*_EDL_/*C*_EDL_). The sensitivity of the sensing platform, as determined from the calibration curve’s gradient, is measured at 4.66 μF/[UZn] (ng/mL), with an *R*^2^ value of 0.98. The LOD for the device, according to eq 4(99,100) is calculated to be 7.33 ng/mL.
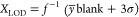
4where the symbol *f*^–1^ represents the inverse function of the calibration linear fitting line, as shown in Figure 3e. The notation *y̅* signifies the mean value of the blank sample, while σ denotes the standard deviation of the blank sample. To prove the selectivity, the modified sensing platform was tested with a mixture of Cu^2+^ and UZn ions. In the modified sensing platform designed to detect UZn, calcium and magnesium, which are the main divalent cations in urine, do not have an affinity to interact with zincon.^54,65^ The pH level is set to 9.8, which is highly specific for the zinc interaction with zincon. As a result, Cu^2+^, the most competitive element, is selected for the selectivity test. A solution with UZn and Cu^2+^ concentrations was introduced to the sensing platform. Based on the results, the device specifically detects UZn, as illustrated in Figure 3f, and the selectivity is clear from the effect of each ion on *C*_EDL_ modulation. Accordingly, the *C*_EDL_ alteration is not significant after adding Cu^2+^. After adding UZn and the mixture of Cu^2+^ + Zn^2+^ solutions, the same value of *C*_EDL_ is achieved as a result of the alkaline nature of the solution which enhances the interaction between zincon and UZn.^65^ This result demonstrates the selectivity when the solution is tested with complex elements in the solution. The repeatability offered valuable information regarding the sensor’s capacity to consistently and accurately detect levels during multiple repetitions. In essence, a reliable sensor should exhibit a consistent response for the same level of target when subjected to multiple repetitions. The test results imply that the modified sensing platforms are reliable for use up to ∼6 times before the sensing sites are destroyed by electrochemical reaction, which is seen as their *C*_EDL_ increases (Figure 3g). The stability of the modified sensing platform results by the EIS technique is thoroughly tested by analyzing the number of UZn tests, as presented in Figure 3h. Based on the results, it is evident that the performance of the modified porous carbon electrode remains stable for up to 15 days. However, this relatively short lifespan may be attributed to air contamination, which blocks the reaction sites and limits the accessibility of UZn to interact with zincon within the porous structure.

In an ideal scenario within the realm of biomedical applications, sensing platforms should have the capability to continuously monitor targets and assess the dynamic concentration of biomarkers over an extended time frame. To implement this approach effectively, a significant challenge arises due to the inherent limitation of single platforms, which struggle to provide multipoint detection capacity.^101^

**Figure 4:**
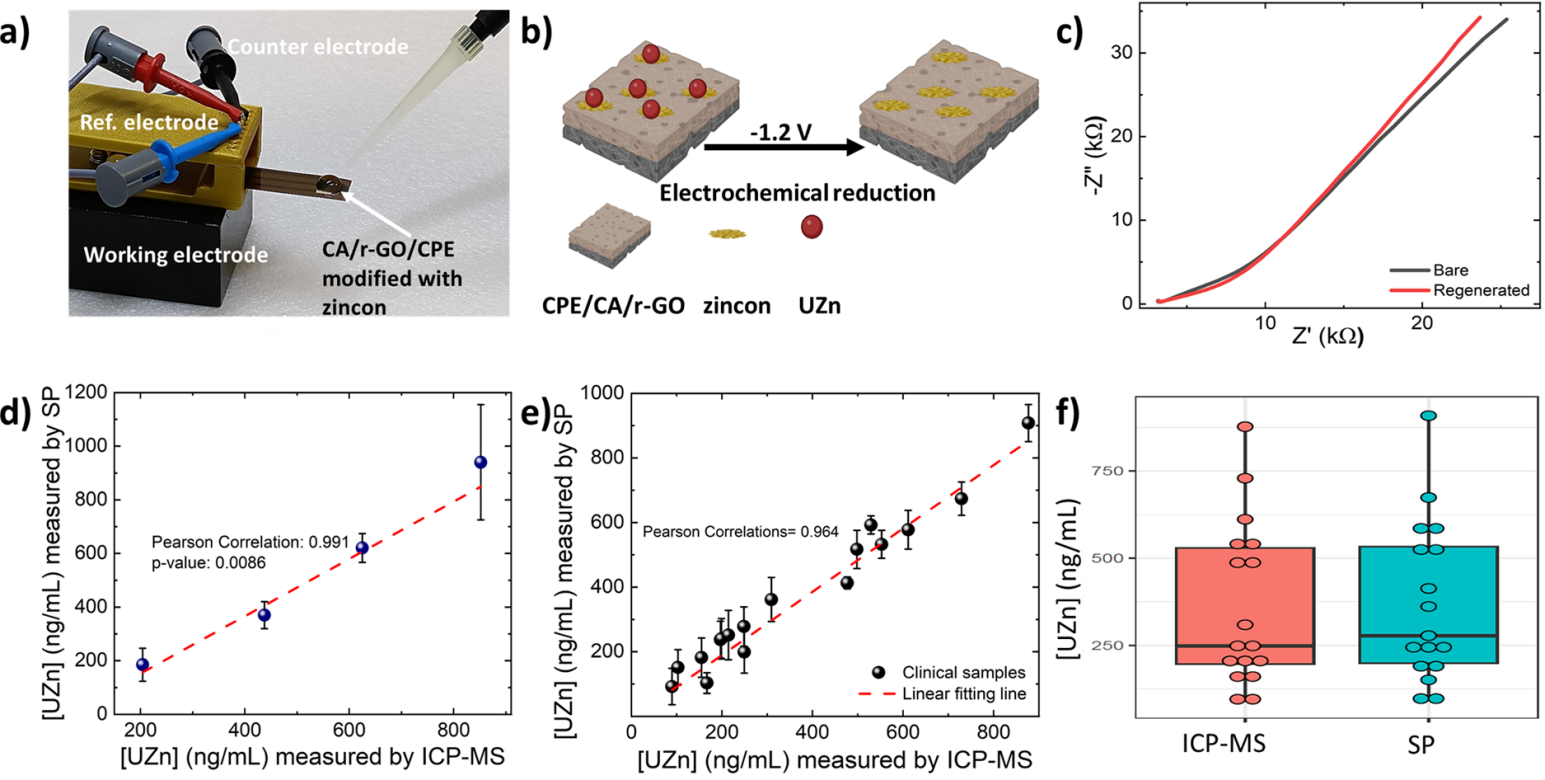
Image of CA/r-GO/SPCE modified with zincon (a), schematic representation of the one-step electrochemical regeneration process on the modified sensing platform (b), and Nyquist plot of the functionalized and regenerated electrode (c). Correlative analysis of measurements obtained from the CBR material (d) and clinical samples (e). Scattered plot depicting the detection of UZn from both ICP-MS and the sensing platform results (f).

Biosensors have a limitation in detecting targets due to the saturation of their surface receptors. Consequently, various techniques for surface cleaning have been devised to rejuvenate the saturated microelectrodes. These processes enable the seamless operation of multipoint detection strategies over an extended duration without requiring replacement of saturated biosensors. Nonetheless, the majority of cleaning techniques necessitate the use of aggressive cleaning solutions, often characterized by either low or high pH levels. Additionally, these methods involve time-consuming procedures that have the potential to harm the surface of the biosensor. Of particular concern is the damage inflicted on the receptors during the regeneration process. Such damage can impair their capacity to effectively capture target(s) of interest, leading to reduced sensitivity and selectivity. Figure 4b illustrates the schematic image of a one-step regeneration strategy; by applying −1.2 V as the reduction potential of zinc for 5 min in 0.05 M PBS (pH 7.4), the sensing platform is regenerated, and the incorporated UZn is detached and subsequently removed from the zincon-modified layer (Figure 4c) via washing with DI water. This unique feature opens avenues for future applications, allowing it to be utilized several times before reaching the end of its operational life. This adaptability makes it suitable for deployment in both disposable and nondisposable settings, vastly expanding its range of applications. Figure 4d presents data from the analysis of CBR samples with a modified sensing platform, which supports the accuracy and selectivity of our modified sensing platform when used with various types of material. From the results obtained from our capacitive system and ICP-MS, an observed correlation coefficient of 0.991 underscores the device’s commendable reliability in diagnosing urinary zinc levels as an indicator of prostate cancer.

### 4.5 Benchmarking Using a Clinical Urine Sample

The performance of the modified sensing platform was formally compared to that of the clinical gold standard method using ICP-MS. Twenty urinary samples were randomly selected for analysis by a modified sensing platform and ICP-MS in parallel. In our case using a modified sensing platform, out of the 20 samples analyzed, our device successfully detected a UZn concentration of less than 1000 ng/mL in 17 of them. In contrast, the remaining three samples exhibited concentrations higher than the upper detection limit of our modified sensing platform. Data comparison from ICP-MS and the modified sensing platform confirm a high Pearson correlation with *R*^2^ = 0.964 (Figure 4e). Statistical results confirm that the modified sensing platform is a promising capacitive system, providing a convenient tool for prostate cancer diagnosis (Figure 4f). The independent *T* test is employed to analyze the correlation and discrepancies in the results obtained from clinical samples measured by both ICP-MS and the sensing platform. The test indicates that there is no significant difference between the two groups (*p*-value = 0.93), and they are well correlated. These results confirm the accuracy of the porous modified sensing platform when real urinary samples are measured. The level of significance for the statistical tests is 0.05. The suggested zincon-modified porous SPCE sensing platform demonstrated the ability to quantify UZn in various sample sources following the precise, user-friendly, highly specific and sensitive, noninvasive, and cost-effective procedure. Upon comparison of its performance with other studies, the zincon-modified porous SPCE sensing platform exhibits significant potential for UZn detection (Table 1). Notably, most studies in Table 1 focused on measuring zinc in blood serum involving invasive sampling procedures. In contrast, the modified porous SPCE sensing platform presented has a low limit of detection (LOD), high specificity, cost-effectiveness, and the crucial ability to regenerate, marking an essential environmental advantage.

**Table 1:** Comparison of Various Studies on the Detection of Zn Electrochemically

surface modification	method of detection	sample	LOD (ng/mL)	range of detection (ng/mL)	ref
Nafion-CNT-gold electrode	anodic stripping voltammetry (ASV)	serum	18 (in buffer solution)	180–2500	(102)
Bismuth-GO	anodic stripping voltammetry (ASV)	seminal fluid	6 (in buffer solution)	20–8000	(103)
polyethyleneimine, poly (sodium 4-styrenesulfonate), and mercury nitrate on carbon fiber	square-wave ASV (SWASV)	blood and urine	9 (in buffer solution)	20–2000	(104)
zincon-exfoliated graphite	differential pulse voltammograms (DPV)	serum	5 (in buffer solution)	250–1500	(38)
zincon-modified CNT	square-wave voltammetry (SWV)	artificial urine and saliva	20 (in artificial urine); 30 (in artificial saliva)	125–1000	(39)
this work	nonfaradaic electrochemical impedance spectroscopy (EIS)	urine	7.33 (in artificial urine)	0.1–1000	

## 5 Conclusions

In summary, we design and develop a label-free, real-time, precise, and cost-effective sensing platform using the modified porous screen-printed carbon electrode in conjugation with the EIS impedimetric technique to detect UZn as a prostate cancer biomarker. The study introduces a groundbreaking approach by integrating label-free impedimetric sensing and strategically introducing porosity through the surface modification techniques of screen-printed carbon electrodes. Utilizing a cellulose acetate and reduced graphene oxide composite, along with external doping of zincon as a receptor, the recognition layer of our capacitive sensing platform is engineered to exhibit enhanced porosity and selectivity, critical for capturing and interacting with UZn with high sensitivity. The portable capacitive system uses an impedimetric approach along with the refined technique of nonfaradaic electrochemical impedance spectroscopy. This is applied at an optimal frequency modulation to detect UZn with high accuracy in artificial urine, clinical biochemistry reference materials, and clinical urine samples. With a detection limit of 7.33 ng/mL, the sensing platform displays high accuracy and selectivity with minimal response to nonspecific elements in artificial urine, thus making it a reliable diagnostic tool. Its reliability is verified from the correlation values of the CBR samples and that of the clinical samples. The clinical samples have a smaller *R*^2^ value, which we believe is a result of additional salts in the urine that partially interfere with the active sites.

## Supporting Information Available

Additional experimental details and methods; SEM images of the sensing platform after CA/r-GO modification and the cross-section image after zincon functionalization; FTIR characterization of CE, which is modified with CA/r-GO, zincon, and enriched with UZn and X-ray diffraction pattern of CA/r-GO, modified sensing platform with zincon; and enriched modified sensing platform with UZn; AFM images of the surface-modified sensing platform with CA/rGO, after zincon functionalization, and after introducing UZn; Bode plot after each step of cleaning and Nyquist plots after zincon modification; ttest statistical analysis; scattered plot depicting the detection of UZn from both ICP-MS and the sensing platform results; and *R*_s_ values after each repetition of EIS measurement (PDF)
