# Supplementary material for: Rapid Near-Patient Impedimetric Sensing Platform for Prostate Cancer Diagnosis
Source: ACS Omega. 2024 Mar 15;9(12):14580–91. doi: 10.1021/acsomega.4c00843 (PMC10976404; doi:10.1021/acsomega.4c00843)
Supplement: Supplementary file 1 — ao4c00843_si_001.pdf [file ao4c00843_si_001.pdf]

Supplementary information

Rapid Near-Patient Impedimetric Sensing Platform for Prostate Cancer Diagnosis

Parisa Dehghani[a], Vaithinathan Karthikeyan[a], Ataollah Tajabadi[a], Dani S. Assi[a],Anthony Catchpole[b], John Wadsworth[b], Hing Y. Leung[c], Vellaisamy A. L. Roy[*d]†*

[a] James Watt School of Engineering, University of Glasgow, Glasgow G12 8QQ, United Kingdom

[b] Scottish Trace Element and Micronutrient Diagnostic and Research Laboratory, Department of Biochemistry, Royal Infirmary, Glasgow, G31 2ER, United Kingdom

[c] Cancer Research UK Scotland Institute, Glasgow, G61 1BD United Kingdom.; School of Cancer Sciences, MVLS, University of Glasgow, Glasgow G61 1BD, United Kingdom.

[d] James Watt School of Engineering, University of Glasgow, Glasgow G12 8QQ, United Kingdom; School of Science and Technology, Hong Kong Metropolitan University, Ho Man Tin, Hong Kong

[*] Corresponding author E-mail: vroy@hkmu.edu.hk

1. Result and Discussion

1.1. Surface morphology characterization:

It is observed in Figure S1, b that the dense layer of CA/r-GO covers the screen-printed carbon electrode's (SPCE) surface. The porous structure is due to fragmentation in polymer chains caused by sonication during the CA/r-GO composite synthesising. Sonication results in the formation and migration of cavitation bubbles inside the polymer solution for some local interactions. The free electrons of the polymer's functional groups interact with delocalized electrons at the surface of the r-GO to generate a pseudo-crosslinking effect [1, 2]. Moreover, by increasing the magnification of the SEM to a higher enlargement (Figure S1a), the dens and homogenous dispersion of the r-GO in the polymeric matrix on the micro and nanoscale is obvious. Also, the porous structure is obvious after zincon functionalization from SEM cross-section image (Figure S1b).

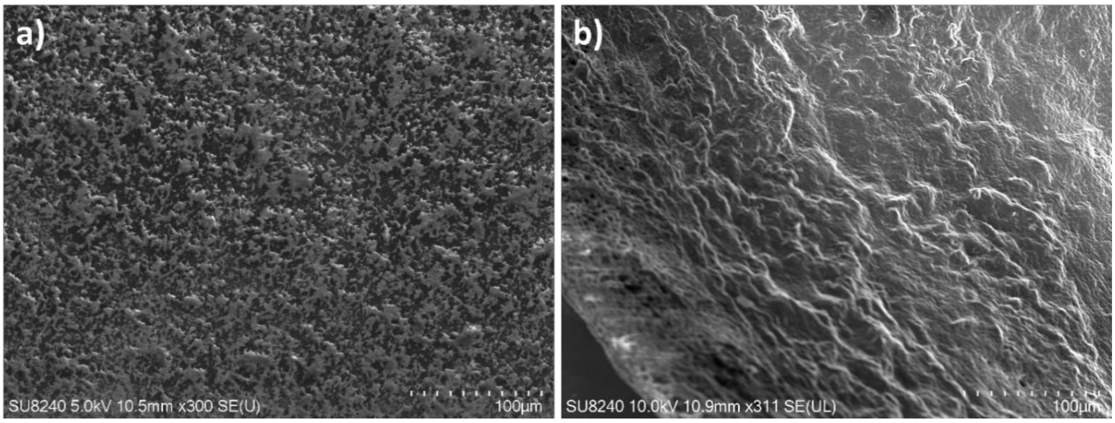

Figure S1. The SEM images of sensing platform after CA/r-GO modification (a), and the cross-section image after zincon functionalization (b).

To further investigate the surface modification and interaction between layers of CA/r-GO functionalized with zincon and UZn sites, we performed Fourier transform infrared (FTIR) spectroscopy (Figure S2a). However, XRD analysis was carried out to identify and demonstrate the structural interaction of the CA/r-GO composite with zincon, and subsequently the modified CA/r-GO/zincon with UZn (Figure S2b).

The presence of broad band peaks observed at 678 cm^{-1} and 1523 cm^{-1} are associated with ≡C-H bonding and C-N deformation indicating the bonds of zincon functionalized CA/r-GO/CE, respectively. The peak located at 1619 cm^{-1} corresponds to C=C bonds, and confirm the π-π interaction between zincon and CA/r-GO/CE, while peaks between 1200-1250 cm^{-1} are attributed to C-O. FTIR peaks at 2844 cm^{-1} and 3275 cm^{-1} correspond to zincon and are associated with C=CH and OH in carboxylic acid group, which may be masked in CE-CA/r-GO-zincon composite layer [3, 4]**.** XRD analysis was carried out to identify and demonstrate the structural interaction of the CA/r-GO composite with zincon, and subsequently the modified

CA/r-GO/zincon with UZn. The observed peak at 26.6˚ represents a highly ordered crystal structure (002) of the graphene with a basal spacing of 3.4 A˚. XRD peak at 9.07˚ represents the basal spacing resulting from oxidation groups of r-GO [5]. It can also be observed that the intensity of these peaks at 38.3˚ decreases as a result of the zincon functionalization of CA/r-GO. The effect of UZn introduced on the porous surface can be clearly seen by the fading of 18.2˚ peak, which corresponds to the (002) crystal surface of carbon (Figure S2b).

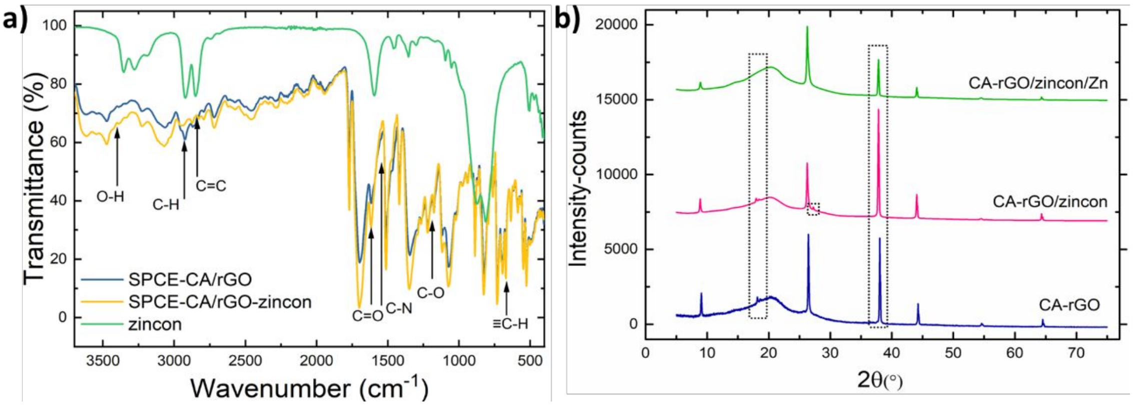

Figure S2. FTIR characterization of CE, which is modified with CA/r-GO, zincon, and enriched with UZn (a), and X-ray diffraction pattern of CA/r-GO (blue line), modified sensing platform with zincon (pink line) and enriched modified sensing platform with UZn (b).

1.2. EIS modified sensing platform performance characterization:

Charge perturbation leads to impedance changes and electrode-electrolyte interface polarization by a small 10 mV input voltage applied to the electrochemical system. Changing the impedance can provide valuable information corresponding to the interaction between the UZn and zincon that are immobilized on the surface of modified sensing platform. Also, by adding zinc, root-mean-square (RMS) was decreased from 9.9 nm after zincon modification to 1.7 nm, as shown in Figure S3, and penetration efficiency reduced with decreasing porosity, according to an AFM test. A decrease in porosity also resulted in a reduction of electrolyte solution penetration inside the zincon layer [6, 7].

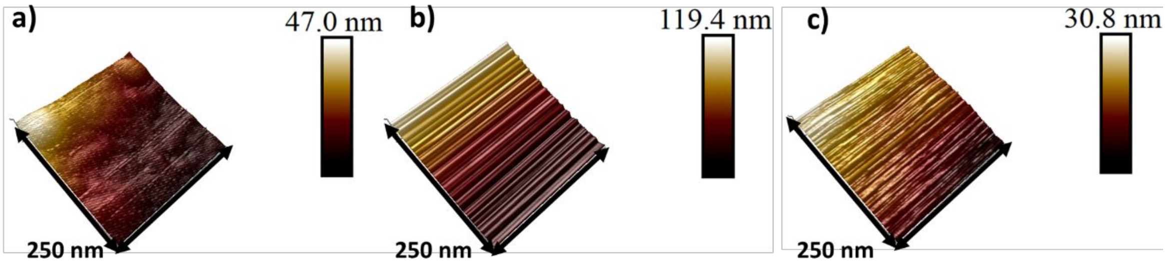

Figure S3. Atomic force microscopy (AFM) images of the surface-modified sensing platform. The root mean square (RMS) roughness is measured at 2.5 nm for the modified CA/r-GO sensing platform (a). Following the introduction of zincon (b) and subsequently UZn (c), the RMS roughness increases to 9.9 nm due to external doping. This augmentation is then followed by a reduction to 1.7 nm, attributed to the interaction between zincon and UZn, which results in decreased penetration efficiency.

1.3. Study the repeatability and stability of modified sensing platform

The repeatability of the sensing platform was analysed by adding constant amount of UZn on the surface of the modified sensing platform for several time and measuring the EIS which is presented in Figure 3h. The graph illustrated that the modified sensing platform could be used 6 times before any significant error. Afterwards, the C_{EDL} reduction can be seen in 7th repeating time. It results from surface damage due to multiple cleaning processes and usages. The C_{Edl} values of each run is presented in table S1.

Table S1. Rs values after each repetition of EIS measurement

Test cycle	**1st**	**2nd**	**3rd**	**4th**	**5th**	**6th**	**7th**
**$\|\Delta C\|$%**	26.47	25.41	25.06	24.02	23.98	23.55	17.12

2. <u>Methodology</u>

2.1. Electrode functionalization with zincon

For immobilization of zincon on a modified CA/r-GO/CPE, 0.01 g of zincon was dissolved in 0.01 g of methyltrioctylammonium chloride and 5 mL ethylenediamine for 20–22 min, then 10 µL of the solution was added on the surface of the modified electrode. Afterwards, the functionalized CA/r-GO/CPE with zincon was washed with DI water to remove additional zincon and stored under water. The modification of each layer on the surface of the CPE was investigated with scanning electron microscopy (SEM), EDX, atomic force microscopy (AFM), and EIS measurement which is illustrated in Figure S 4b.

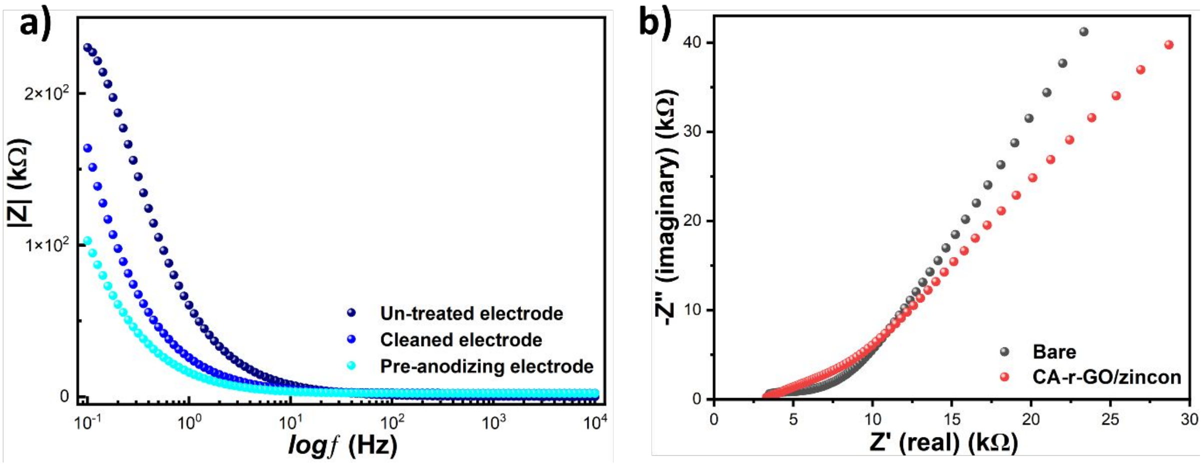

Figure S4. The Bode plot after each step of cleaning and Nyquist plots after zincon modification (b).

2.2. Zn detection in artificial urine

The artificial urine is prepared according to DIN EN1616:1999 standard procedure. DIN EN 1616 specifies the method to test sterile urethral catheters. Here, urea (25 g/L), NaCl (9 g/L), Disodium Hydrogen Orthophosphate, anhydrous (2.5 g/L), Potassium Dihydrogen Orthophosphate (2.5 g/L), NH_3Cl (3g/L), creatinine (2g/L), and sodium sulphite (3 g/L) are weighted and mixed together under continuous stirring.

To storage the artificial urine, the solution is frozen to avoid bacteria growth. Artificial urine samples were prepared that consisted of a variety of salts, including sodium chloride, disodium hydrogen orthophosphate, potassium dihydrogen phosphate, ammonium, creatinine etc (urine (EN 1616:1999)).

2.3. Urine samples preparation

Twenty-one urine samples from the Beatson cancer research institute are aliquoted into 5 smaller 4 mL sections and stored in a -80 ˚C freezer for future usage. Each sample is thawed and pipetted before being transferred to a 10 mL centrifuge tube. Urine samples are transferred to the tube via a syringe filter to filter out the solid phase of samples. The samples are centrifuged at 3000 rpm for 5 min, then aliquoted to 4 sections (1 mL) and stored in a -20 ˚C freezer. One section is transferred to the Scottish Trace Element and Micronutrient Diagnostic and Research Laboratory to trace and measure the UZn level by ICP-MS, and the second section is used for UZn detection by our modified sensing platform.

2.4. Selectivity test

100 ng/mL of Cu^{2+} solution is prepared, and the pH of the solution is adjusted to 9.8. 100 μL of solution is introduced on the surface of the electrode at 25 ˚C. The DI water is used as a control sample. The mixture of Cu^{2} and Zn^{2+} solution with the same concentration of 100 ng/mL at pH 9.8 are prepared and applied to the surface of the modified sensing platform to investigate the selectivity performance of the biosensor via the EIS technique.

2.5. Sensitivity curve equation and unknown sample diagnosis

This analysis is performed with UZn samples in artificial urine and CBR reference material samples. EIS technique is used to study the performance of the modified sensing platform. The experiment is done four times for the CBR samples and the average results for C_{Edl} is analysed. The C_0 is the impedance value of the pure sample without UZn. The sensitivity curve of $|\Delta C_{Edl}|$ (%) with the baseline ($100*\Delta C_{Edl}/C_{Edl}$) is plotted to measure the concentration of unknown samples by exclusively replacing the C_{Edl} values in the equation to obtain the level of UZn in unknown clinical samples. The calibration curve and results obtained from various Lab-based UZn samples and CBR reference material are in good agreement overall.

2.6. Reusability and repeatability of the biosensor

Stability and repeatability are important elements as they can confirm the performance of the biosensor through repeated measurements. Testing these parameters is done for the modified sensing platform after immobilization of zincon on the surface of the SPCE electrode by applying 100 ng/mL UZn solution. For repeatability, 100 μL of UZn solution is pipetted on the surface of the modified SPCE electrode, and the EIS technique is applied. For stability, the sensor is kept at room temperature for one month, and its performance is investigated each week via EIS after introducing a 100 ng/mL UZn solution on a modified SPCE electrode. For each experiment, the data is repeated 3 times.

2.7. Statistical analysis

The T-test is employed to analyze the correlation and discrepancies in the results obtained from clinical samples measured by both ICP-MS and the sensing platform. The test reveals a no significant difference between the two groups (p-value = 0.93), indicating a strong correlation.

These findings validate the accuracy of the porous modified sensing platform when dealing with real urinary samples. The level of significance for the statistical tests is set at 0.05.

References:

1. Ioniță, M.; Crică, L. E.; Voicu, S. I.; Dinescu, S.; Miculescu, F.; Costache, M.; Iovu, H., Synergistic effect of carbon nanotubes and graphene for high performance cellulose acetate membranes in biomedical applications. *Carbohydrate polymers* **2018,** *183*, 50-61.
2. Thakur, V. K.; Voicu, S. I., Recent advances in cellulose and chitosan based membranes for water purification: A concise review. *Carbohydrate polymers* **2016,** *146*, 148-165.
3. Salehirozveh, M.; Dehghani, P.; Zimmermann, M.; Roy, V. A.; Heidari, H., Graphene field effect transistor biosensors based on aptamer for amyloid-β detection. *IEEE Sensors Journal* **2020,** *20* (21), 12488-12494.
4. Teng, Y.; Singh, C. K.; Sadak, O.; Ahmad, N.; Gunasekaran, S., Electrochemical detection of mobile zinc ions for early diagnosis of prostate cancer. *Journal of Electroanalytical Chemistry* **2019,** *833*, 269-274.
5. Guo, X.; Kulkarni, A.; Doepke, A.; Halsall, H. B.; Iyer, S.; Heineman, W. R., Carbohydrate-based label-free detection of Escherichia coli ORN 178 using electrochemical impedance spectroscopy. *Analytical chemistry* **2012,** *84* (1), 241-246.
6. Salehirozveh, M.; Kure Larsen, A. K.; Stojmenovic, M.; Thei, F.; Dong, M., In-situ PLL-g-PEG Functionalized Nanopore for Enhancing Protein Characterization. *Chemistry–An Asian Journal* **2023,** *18* (17), e202300515.
7. Xu, A.; Weng, Y.; Zhao, R., Permeability and equivalent circuit model of ionically conductive mortar using electrochemical workstation. *Materials* **2020,** *13* (5), 1179.
8. Hernández, H. H.; Reynoso, A. R.; González, J. T.; Morán, C. G.; Hernández, J. M.; Ruiz, A. M.; Hernández, J. M.; Cruz, R. O., Electrochemical impedance spectroscopy (EIS): A review study of basic aspects of the corrosion mechanism applied to steels. *Electrochemical Impedance Spectroscopy* **2020**, 137-144.
